# Risk of Stroke Following Herpes Zoster: A Self-Controlled Case-Series Study

**DOI:** 10.1093/cid/ciu098

**Published:** 2014-04-02

**Authors:** Sinéad M. Langan, Caroline Minassian, Liam Smeeth, Sara L. Thomas

**Affiliations:** Faculty of Epidemiology and Population Health, London School of Hygiene and Tropical Medicine, United Kingdom

**Keywords:** herpes zoster, stroke, self-controlled case-series study

## Abstract

***Background.*** Herpes zoster is common and vaccine preventable. Stroke risk may be increased following zoster, but evidence is sparse and could be explained by differences between people with and without zoster. Our objective was to determine if stroke risk is increased following zoster.

***Methods.*** Within-person comparisons were undertaken using the self-controlled case-series method and data from the UK Clinical Practice Research Datalink (1987–2012). Participants had a first-ever diagnosis of zoster and stroke within the study period. Stroke incidence in periods following zoster was compared with incidence in other time periods. Age-adjusted incidence ratios (IRs) and 95% confidence intervals (CIs) were calculated.

***Results.*** A total of 6584 individuals were included. Stroke rate was increased following zoster compared with the baseline unexposed period, then gradually reduced over 6 months: weeks 1–4 (age-adjusted IR, 1.63; 95% CI, 1.32–2.02), weeks 5–12 (IR, 1.42; 95% CI, 1.21–1.68), and weeks 13–26 (IR, 1.23; 95% CI, 1.07–1.42), with no increase thereafter. A stronger effect was observed for individuals with zoster ophthalmicus, rising to a >3-fold rate 5–12 weeks after zoster. Oral antivirals were given to 55% of individuals: IRs for stroke were lower among those receiving antivirals compared with those not treated, suggesting a protective effect.

***Conclusions.*** We have established an increased stroke rate within 6 months following zoster. Findings have implications for zoster vaccination programs, which may reduce stroke risk following zoster. The low antiviral prescribing rate needs to be improved; our data suggest that antiviral therapy may lead to a reduced stroke risk following zoster.

## Abstract (precis)

Adults developing zoster are at increased risk of stroke for 6 months with a >3-fold increased risk following zoster ophthalmicus. Incidence ratios of stroke were lower among those receiving antiviral drugs compared with untreated individuals, suggesting a possible protective effect.

**(See the Editorial Commentary by Nagel and Gilden on pages 1504–6.)**

There is mounting epidemiological evidence from different populations using different study designs of strong associations between acute systemic infections and risk of acute vascular events [1, 2]. It is believed that the transient increase in risk relates to endothelial dysfunction on a background of arteriopathy with plaque rupture and hypercoagulability [3]. The effect of specific infections on acute vascular events including stroke has been less explored.

Herpes zoster is a significant public health problem in aging populations, affecting 1 million Americans per year and >88 650 immunocompetent people aged >60 years annually in the United Kingdom and resulting in significant complications [4–6]. It occurs following reactivation of latent varicella zoster virus (VZV) infection. The initial presentation consists of a painful vesicular rash that can lead to prolonged pain and postherpetic neuralgia (PHN), with a major impact on quality of life [5]. The importance of zoster and associated complications are reflected by the introduction of major adult vaccination programs in the United States, United Kingdom, and elsewhere to prevent disease.

In addition to systemic inflammation after the acute reactivation of dormant VZV infection, zoster could increase stroke risk by viral invasion of arterial walls and induction of vasculopathy [7]. Case reports have reported strokes following zoster [8, 9]. Two cohort studies of adults from Taiwan showed a 1.3-fold (95% confidence interval [CI], 1.1- to 1.6-fold) and 4.5-fold (95% CI, 2.5- to 8.3-fold) increased risk of stroke in the year following zoster and herpes zoster opthalmicus, respectively [10, 11]. However, effect estimates were not adjusted for key confounders including body mass index and atrial fibrillation [12]. It could be that the observed increased risk of stroke following zoster may be entirely due to residual confounding. Thus, rigorous epidemiological studies that deal with confounding are needed to determine if there is an increased risk of stroke following zoster. Such an effect, if genuine, would have important implications for targeting zoster vaccination programs. We set out to determine if adults experiencing zoster at any site or herpes zoster ophthalmicus are at increased risk of stroke within 12 months after zoster using a novel method that inherently avoids between-person confounding, the self-controlled case series (SCCS) method. We also examined whether the effect was modified by receipt of antiviral therapy.

## METHODS

### Ethics Approval

Ethics approval was obtained from the Independent Scientific Advisory Committee of the Clinical Practice Research Datalink (CPRD; formerly GPRD) and the Ethics Committee of the London School of Hygiene and Tropical Medicine.

### Data Source

The CPRD is the world's largest computerized database of anonymized longitudinal patient records from general practice, containing data on approximately 8% of the UK population. Data are obtained from >625 general practices for a currently registered population of >5 million people representative of the general UK population [13]. The data contain complete medical records including prescriptions and feedback from referrals and hospitalizations. CPRD data have been widely used for epidemiological research, and studies confirm the validity of stroke diagnoses in this data source [14–17]. A proportion of individuals in CPRD are linked to Hospital Episode Statistics (HES), containing details of admissions to National Health Services hospitals in England. The HES-CPRD link provides information on patients hospitalized from 1997 onward.

### Study Design

**Figure 1.**
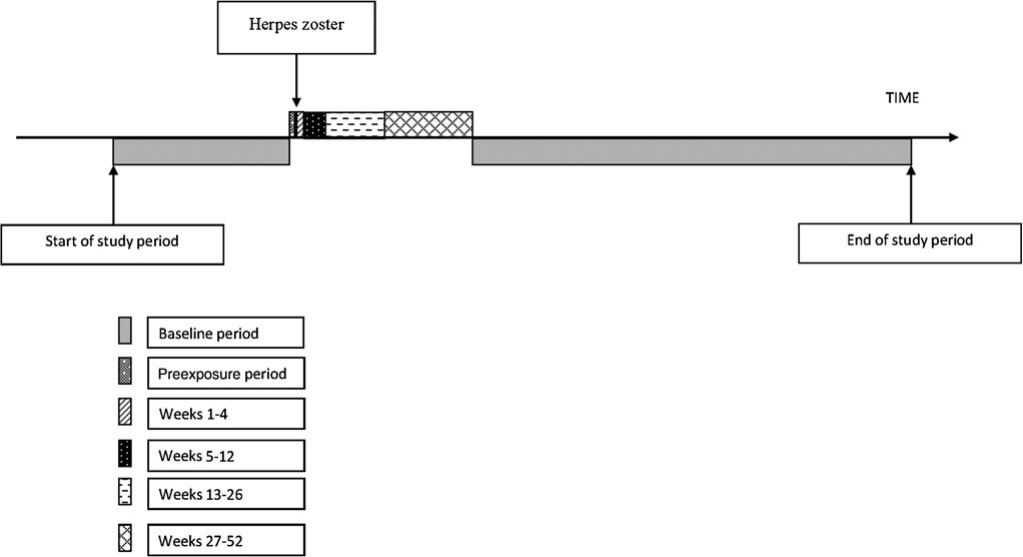
Pictorial representation of the self-controlled case series study.

This study estimated stroke risk following an acute episode of zoster. Critical differences between those who develop and do not develop zoster are difficult to capture in many observational study designs, leading to residual confounding. The SCCS method compares risks during different time periods within individuals. Thus, risks are compared in the period following zoster to other “unexposed” time periods (Figure 1). The major advantage of SCCS for our research question is that it avoids between-person confounding (due to differing stroke risk among individuals with and without zoster) [18]. The null hypothesis was that stroke risk is not increased following zoster infection.

### Participants

All adults (aged ≥18 years) with evidence of incident zoster and an incident stroke were identified. Follow-up began at the later of the date that an individual registered with a practice or the date that the individual's practice met established quality standards. Follow-up ended when the individual left his/her practice or died, or the practice left the database. To ensure observation of incident rather than prevalent or past zoster and stroke, the study period (observation period for incident events) began 12 months after the start of follow-up [19]. To restrict to first-ever episodes of incident zoster and stroke, we excluded individuals with evidence of zoster, PHN, or stroke before the study period. As our major interest was in incident arterial strokes, we additionally excluded individuals whose incident episode was a transient ischemic attack (TIA) and those with subarachnoid hemorrhage or with specific established risk factors for subarachnoid hemorrhage, such as cerebral aneurysms in the circle of Willis or arteriovenous malformations. Zoster can occasionally lead to cerebral aneurysms by damaging vessel walls; hence, individuals with nonspecific cerebral aneurysms were not excluded from the study [7]. Individuals with a record of encephalitis within 12 months after stroke were excluded, as these may represent encephalitis initially misdiagnosed as stroke.

### Exposure and Outcome

First-ever incident zoster and arterial stroke were identified from CPRD and, in those with linked data, from hospital record primary diagnoses using Read and *International Classification of Diseases, Tenth Revision* (*ICD-10*) diagnostic codes, respectively. Herpes zoster ophthalmicus was identified from the presence of specific diagnostic codes, or, when the zoster codes were nonspecific, from diagnoses of or treatments for acute eye infection within 2 weeks of zoster onset or from records of first-ever specific nonacute eye conditions known to be associated with zoster (eg, conjunctival scarring or episcleritis), within 3 months after zoster onset. Oral antiviral treatments were identified from therapy records 1 week before and up to 2 weeks after incident zoster.

Stroke episodes were created whereby diagnostic codes for stroke or TIA within 28 days of each other were considered part of the same episode. This method facilitated differentiation of the type of stroke (cerebral infarction, hemorrhagic stroke, or stroke of unspecified type) and between stroke and TIA (when all records in an episode were TIA), for later exclusion of TIA. An important assumption of the SCCS method is that recurrent outcome events must be independent, that is, the occurrence of 1 event must not alter the probability of a subsequent event occurring. Because having 1 stroke may increase the risk of a further stroke, only the date of onset of the first stroke for each individual was included in the study [20].

### Analysis

The exposed period started the day after incident zoster and extended to 12 months, subdivided into weeks 1–4, 5–12, 13–26, and 27–52 following zoster. All other observation time made up the baseline (unexposed) period, with the exception of the day of zoster (stroke records on the same day as incident zoster may be retrospective diagnoses [21]) and the 4-week period prior to zoster (the chance of developing and presenting with zoster after an incident stroke may be different from other time periods; hence, stroke incidence during this time may be lower or higher than during baseline). The primary analyses assessed the effect of (1) zoster at any site and (2) zoster ophthalmicus on stroke (all types). Additional analyses were undertaken for each stroke type separately (cerebral infarctions, hemorrhagic, and type-unspecified strokes). Conditional Poisson regression was used to calculate incidence ratios (IRs) and 95% confidence intervals (CIs) for stroke within each stratum of the exposed period compared with baseline, adjusting for age in 5-year bands, with a sensitivity analysis adjusting for 2-year age bands. A further stratified analysis was undertaken to determine if stroke risk following zoster was different between those who received and those who did not receive antiviral treatment for zoster.

When using the SCCS method, the occurrence of an event must not alter the probability of subsequent exposure and must not censor the observation period. Risk of death is increased following a stroke, removing the possibility of being exposed to zoster and censoring the individual's follow-up. Hence, a sensitivity analysis was undertaken excluding individuals who died or whose follow-up ended within 90 days of their stroke. Further sensitivity analyses were undertaken to include the day of zoster in the exposed period, and to restrict to individuals aged ≥60 years and to patients whose records were linked to HES (in which date of stroke onset may be better recorded). Analyses were undertaken using Stata (version 12.0).

## RESULTS

Of the 11 997 individuals identified with first-ever zoster and first-ever stroke or TIA within the study period, 6584 fulfilled study inclusion criteria (Supplementary Figure 1). The median age at stroke was 77 years (interquartile range [IQR], 69–84 years), and 57% were women. The median observation period was 12.5 years (IQR, 8.7–17.1 years). Most strokes (n = 3944 [60%]) were of unspecified type, with smaller numbers having records confirming ischemic (n = 2174 [33%]) and hemorrhagic (n = 422 [6%]) strokes. Most cases had zoster of an unspecified site (n = 6126 [93%]), 426 (6%) had evidence of herpes zoster ophthalmicus, and 32 (0.5%) had zoster in other branches of the trigeminal nerve (Table 1). Systemic antiviral therapy was received in 3647 (55%) study participants.

**Table 1.** Characteristics of 6584 Eligible Patients With Both Zoster and Stroke During the Study Period

Characteristic	All Zoster Cases (N = 6584)	Zoster Site		
		Ophthalmic (n = 426)	Other Trigeminal (n = 32)	Site Unspecified (n = 6126)
Age at index stroke^a^, y, median (IQR)	77.0 (68.9–83.8)	78.9 (70.8–84.8)	73.0 (64.6–78.6)	76.9 (68.7–83.7)
Male sex, No. (%)	2813 (42.7)	189 (44.4)	23 (71.9)	2601 (42.5)
Total observation, y, median (IQR)^b^	12.5 (8.7–17.1)	12.0 (8.5–16.8)	13.9 (8.3–18.5)	12.5 (8.7–17.2)
Eligible for HES, No. (%)	3617 (54.9)	239 (56.1)	15 (46.9)	3363 (54.9)
Type of index stroke, No. (%)				
Infarct	2174 (33.0)	145 (34.0)	13 (40.6)	2016 (32.9)
Hemorrhagic	422 (6.4)	21 (4.9)	0 (0)	401 (6.6)
Type unspecified	3944 (59.9)	256 (60.1)	19 (59.4)	3669 (59.9)
Other^c^	44 (0.7)	4 (0.9)	0 (0)	40 (0.6)

Abbreviations: HES, Hospital Episode Statistics; IQR, interquartile range.

^a^ First stroke episode in study period.

^b^ Follow-up during study period.

^c^ Stroke episode contained both an infarct and a hemorrhagic stroke code.

The overall rate of strokes was significantly increased in weeks 1–4 postzoster compared with baseline (IR, 1.63; 95% CI, 1.32–2.02) with a slowly diminishing increased rate extending up to 6 months postzoster: weeks 5–12 (IR, 1.42; 95% CI, 1.21–1.68), and weeks 13–26 (IR, 1.23; 95% CI, 1.07–1.42) (Tables 2 and 3). An even stronger effect was observed among individuals not treated with antiviral therapy (weeks 1–4: IR, 2.14; 95% CI, 1.62–2.84), with a similar pattern of resolution (*P* value for interaction = .03) (Table 4). Estimates in weeks 1–4 were nearly double in those not receiving antiviral therapy compared with those receiving treatment. Among individuals treated with oral antivirals, the only period with increased rate of stroke compared with baseline was the 5–12 weeks postzoster (IR, 1.28; 95% CI, 1.02–1.62).

**Table 2.** Age-Adjusted Incidence Ratios for Stroke in Risk Periods Following Zoster

Outcome and Risk Period	No. of Cases	IR^a^ (95% CI)
Stroke (all types)	6584	
Risk period after zoster		
1–4 wk	90	1.63 (1.32–2.02)
5–12 wk	149	1.42 (1.21–1.68)
13–26 wk	215	1.23 (1.07–1.42)
27–52 wk	303	0.99 (.88–1.12)

Abbreviations: CI, confidence interval; IR, incidence ratio.

^a^ Incidence ratio, adjusting for age in 5-year bands.

Analyses by zoster site indicated that herpes zoster ophthalmicus was associated with a stronger effect on stroke overall (weeks 5–12: IR, 3.38; 95% CI, 2.18–5.24 for herpes zoster ophthalmicus and IR, 1.30; 95% CI, 1.09–1.5 for those with site unspecified) with nonoverlapping confidence intervals. For herpes zoster ophthalmicus, the most marked increase was delayed until weeks 5–12. Similar findings were observed when combining individuals with zoster in ophthalmic and other branches of the trigeminal nerve (Table 3).

**Table 3.** Age-Adjusted Incidence Ratios for Stroke in Risk Periods Following Zoster, by Site of Zoster

Site of Zoster and Risk Period	No. of Cases	IR^a^ (95% CI)
Ophthalmic	426	
Risk period post zoster		
1–4 wk	6	1.82 (.81–4.10)
5–12 wk	22	3.38 (2.18–5.24)
13–26 wk	15	1.39 (.83–2.35)
27–52 wk	16	0.82 (.49–1.36)
Ophthalmic/other trigeminal	458	
Risk period postzoster		
1–4 wk	6	1.74 (.77–3.91)
5–12 wk	22	3.23 (2.08–4.99)
13–26 wk	16	1.41 (.85–2.33)
27–52 wk	18	0.87 (.54–1.41)
Site unspecified	6126	
Risk period postzoster		
1–4 wk	84	1.62 (1.30–2.02)
5–12 wk	127	1.30 (1.09–1.55)
13–26 wk	199	1.22 (1.06–1.41)
27–52 wk	285	1.00 (.89–1.13)

Abbreviations: CI, confidence interval; IR, incidence ratio.

^a^ Incidence ratio adjusting for age in 5-year bands.

Stratifying herpes zoster ophthalmicus analyses by receipt of oral antivirals also suggested a stronger effect among individuals who did not receive antiviral treatment, with a >5-fold increase in the rate of stroke during weeks 5–12 postherpes zoster ophthalmicus (IR, 5.47; 95% CI, 2.80–10.71). A less marked (though statistically significant) effect was observed among those receiving antiviral therapy for herpes zoster ophthalmicus (IR, 2.57; 95% CI, 1.43–4.62, *P* value for interaction = .33) (Table 4).

**Table 4.** Age-Adjusted Incidence Ratios for Stroke in Risk Periods Following Zoster, Stratified by Oral Antiviral Drug Prescriptions

Site of Zoster and Risk Period	Oral Antiviral Prescription^a^		No Oral Antiviral Prescription	
	No. of Cases	IR^b^ (95% CI)	No. of Cases	IR^b^ (95% CI)
Zoster (all)	3647		2937	
Risk period post zoster				
1–4 wk	38	1.23 (.89–1.71)	52	2.14 (1.62–2.84)
5–12 wk	75	1.28 (1.02–1.62)	74	1.61 (1.27–2.03)
13–26 wk	117	1.19 (.99–1.44)	98	1.29 (1.05–1.58)
27–52 wk	184	1.08 (.93–1.25)	119	0.89 (.74–1.07)
Ophthalmic	299		127	
Risk period post zoster				
1–4 wk	3	1.26 (.40–3.96)	3	3.27 (1.02–10.44)
5–12 wk	12	2.57 (1.43–4.62)	10	5.47 (2.80–10.71)
13–26 wk	12	1.55 (.86–2.79)	3	1.00 (.31–3.18)
27–52 wk	10	0.70 (.37–1.33)	6	1.12 (.48–2.59)
Site unspecified	3337		2789	
Risk period post zoster				
1–4 wk	35	1.23 (.88–1.73)	49	2.10 (1.58–2.81)
5–12 wk	63	1.17 (.91–1.51)	64	1.45 (1.13–1.87)
13–26 wk	105	1.16 (.96–1.42)	94	1.29 (1.05–1.59)
27–52 wk	172	1.10 (.94–1.29)	113	0.89 (.73–1.07)

Abbreviations: CI, confidence interval; IR, incidence ratio.

^a^ *P* values for interaction: zoster overall, *P* = .03; ophthalmic zoster, *P* = .33; and site unspecified zoster, *P* = .03.

^b^ Incidence ratio adjusting for age in 5-year bands.

Similar increased rates of stroke following zoster were observed for arterial ischemic and hemorrhagic stroke (Supplementary Table 1). Sensitivity analyses including the day of zoster in the exposed period, and including only individuals aged ≥60 years, those whose follow-up ended within 90 days following their stroke (possibly indicating death due to stroke), or those whose records were linked to HES did not modify study findings (data not shown). Sensitivity analysis using 2-year age bands did not modify study findings.

## DISCUSSION

The key study finding was that acute zoster is associated with an increased risk of stroke in the first 6 months following zoster. Risk of incident stroke following zoster was higher for individuals with herpes zoster ophthalmicus or other zoster in the distribution of the trigeminal nerve. Results also indicated that the use of oral antiviral therapy to treat acute zoster was associated with a less marked increase in incident strokes following zoster exposure. These findings from a large population-based data source provide important insights into the temporality and magnitude of stroke risk increase following zoster.

Two previous cohort studies from Taiwan reported a 30% increased risk of stroke in the year following zoster with a >4-fold increased risk following herpes zoster ophthalmicus [10, 11]. Neither study adequately addressed key confounders, for example, BMI and atrial fibrillation. In addition, these studies did not assess the timing of increased risk following acute zoster. A recent Danish registry cohort study identified increased stroke risks in the first year following zoster, particularly within 2 weeks of diagnosis. However, that study had important limitations, including using antiviral treatment as a proxy for zoster diagnoses and inadequate control for confounding. Hence, the study assessed the effect of *treated zoster* on stroke; also, the exposed group would include off-label prescribing for herpes simplex virus whereas the unexposed group would include untreated individuals with zoster [22]. A UK cohort study reported increased risks of TIA and of stroke in adults of all ages and in those aged 18–40 years, respectively, during up to 24 years of follow-up following zoster [5]. This study used a cohort design, and the results could be prone to residual confounding due to between-person differences. Additionally, the research question focused on long-term rather than acute effects of zoster on stroke.

[11]. Our study has shown a significantly increased risk of stroke following zoster, in particular for zoster in the trigeminal nerve distribution. Use of the SCCS method is a unique approach; its major advantage for this study question is that fixed confounders are implicitly controlled for, as analyses are within-person. Our findings have demonstrated that the first 3 months postzoster is the key period of increased risk, with resolution over the subsequent 3 months.

The biological mechanisms underlying the observed increased risk of stroke following zoster are likely to be multifactorial. First, inflammation associated with systemic infection may lead to endothelial dysfunction accompanied by disruption of atheromatous plaques and hypercoagulability [23]. In addition, VZV vasculopathy, whereby the VZV virus spreads along nerve fibers and directly involves the vessels, is highly likely to be an important mechanism [7]. In support of the role of VZV vasculopathy is the stronger association between zoster in the trigeminal nerve and the development of arterial stroke. VZV vasculopathy could plausibly trigger either ischemic or hemorrhagic stroke, with the latter arising as a result of arterial dissection and aneurysm following vessel wall damage. In our study, stroke risk was increased overall with similar increases in risk observed for ischemic and hemorrhagic stroke. Our data suggest a slightly delayed effect for herpes zoster ophthalmicus compared with zoster of unspecified sites; this finding is unlikely to be explained by stroke type (Table 1) and may be explained by small numbers in the herpes zoster ophthalmicus group, as there is no obvious biological reason for this difference.

The low antiviral prescription rates observed in this study are consistent with work previously undertaken by our group in this population [24]. Antiviral therapy may be critically important as our study suggests that stroke risk after zoster is lower in those treated with antiviral therapy than in untreated individuals, although this did not reach statistical significance in the smaller subset of ophthalmic zoster patients. The actual difference in risk between those prescribed and those not prescribed antiviral drugs is likely to be even greater than that we observed—individuals given antiviral therapy are likely to have had more severe disease. This differential prescribing of antiviral drugs could introduce confounding by indication, reducing the perceived benefit of antiviral therapy. It is known that antiviral drugs reduce acute pain and zoster severity, accelerate healing. and may reduce PHN; hence, it follows that antiviral drugs might have the potential to reduce other postzoster adverse events, including vascular events, by reducing inflammation [25, 26].

Using the SCCS method, we studied stroke risk following zoster within individuals comparing the risk in exposed periods following zoster to the risk during unexposed periods; thus, important differences in baseline risk of stroke between participants were removed. We controlled for the time-varying effect of age; residual confounding could only occur if other time-varying covariates were strongly associated with the timing of zoster and stroke in many of our study population. The observed increase in risk of stroke following zoster that rose acutely and tailed off over time is highly suggestive of a causal relationship. Our study is a large population-based cohort that is reasonably representative of the general UK population, and the size of the cohort gave increased statistical power. In addition, high-quality data were available on clinical encounters.

CPRD data are routine data captured during clinical care of patients and are not collected to answer a specific research question, so some misclassification of exposures and outcomes is possible. However, this misclassification is likely to be random, and any bias will have tended to underestimate effects.

Most patients in our study (60%) had no information documenting stroke type; despite the large sample size, this reduced the power of our study to assess the effect of zoster on stroke type, although the majority of strokes in adults are likely to be ischemic in nature. A previous CPRD validation study of stroke diagnoses demonstrated that nearly in 90% of individuals with stroke, diagnoses were confirmed reviewing their written medical records, and stroke incidence rates were similar to other sources [27, 28]. Zoster has a highly characteristic clinical presentation and is readily diagnosed by general practitioners; previous studies have reported positive predictive values of >90% for zoster diagnoses in administrative and medical record data [29, 30].

Despite efforts to improve capture of herpes zoster ophthalmicus by looking for acute eye diseases or specific therapy within 2 weeks of zoster and specified nonacute eye diseases within 3 months of zoster, some misclassification of herpes zoster ophthalmicus is likely [31]. The small numbers of individuals we identified with herpes zoster ophthalmicus limited power to assess the association between herpes zoster ophthalmicus and stroke, reducing effect estimate precision.

In conclusion, our study findings have demonstrated an increased risk of stroke in the first 6 months following acute zoster. The risk was higher in individuals with herpes zoster ophthalmicus. The relatively low prescribing rates of antiviral treatment need to be improved, as our data suggest that oral antiviral therapy may lead to a reduction in stroke risk following zoster. Study findings have important implications for zoster vaccination programs, which, in addition to reducing incident zoster and PHN, might have the potential to reduce incident stroke following zoster.

## Supplementary Data

Supplementary materials are available at *Clinical Infectious Diseases* online (http://cid.oxfordjournals.org). Supplementary materials consist of data provided by the author that are published to benefit the reader. The posted materials are not copyedited. The contents of all supplementary data are the sole responsibility of the authors. Questions or messages regarding errors should be addressed to the author.

Supplementary Data
