# Supplementary material for: Risk of Stroke Following Herpes Zoster: A Self-Controlled Case-Series Study
Source: Clin Infect Dis. 2014 Apr 2;58(11):1497–503. doi: 10.1093/cid/ciu098 (PMC4017889; doi:10.1093/cid/ciu098)
Supplement: Supplementary Data [file supp_58_11_1497__index.html]

Risk of Stroke Following Herpes Zoster: A Self-Controlled Case-Series Study — Risk of Stroke Following Herpes Zoster: A Self-Controlled Case-Series Study — Supplementary Data 

# Risk of Stroke Following Herpes Zoster: A Self-Controlled Case-Series Study

## Supplementary Data

Supplementary Data

**Files in this Data Supplement:**

- Supplementary Data - Pdf file
