# Supplementary material for: Risk of Stroke Following Herpes Zoster: A Self-Controlled Case-Series Study
Source: Clin Infect Dis. 2014 Apr 2;58(11):1497–503. doi: 10.1093/cid/ciu098 (PMC4017889; doi:10.1093/cid/ciu098)

E-Table 1. Age-adjusted incidence ratios for stroke sub-types in risk periods following zoster

**Outcome and risk period**	**No. cases**	**IR (95% CI)**
**Type unspecified strokes**	**3944**	
Risk period post zoster		
1-4 wk	58	1.66 (1.28-2.16)
5-12 wk	97	1.47 (1.20-1.81)
13-26 wk	140	1.29 (1.09-1.54)
27-52 wk	185	0.99 (0.85-1.15)
**Cerebral infarcts**	**2174**	
Risk period post zoster		
1-4 wk	26	1.61 (1.09-2.37)
5-12 wk	38	1.20 (0.87-1.66)
13-26 wk	59	1.09 (0.84-1.41)
27-52 wk	92	0.95 (0.77-1.18)
**Haemorrhagic strokes**	**422**	
Risk period post zoster		
1-4 wk	6	1.70 (0.73-3.97)
5-12 wk	13	2.05 (1.17-3.61)
13-26 wk	16	1.53 (0.91-2.55)
27-52 wk	23	1.25 (0.81-1.93)

Note that 44 cases whose stroke episode contained both an infarct and a haemorrhagic code are not included.

*Incidence ratio adjusting for age in 5-year bands

E- Figure 1. Flow diagram of study participants

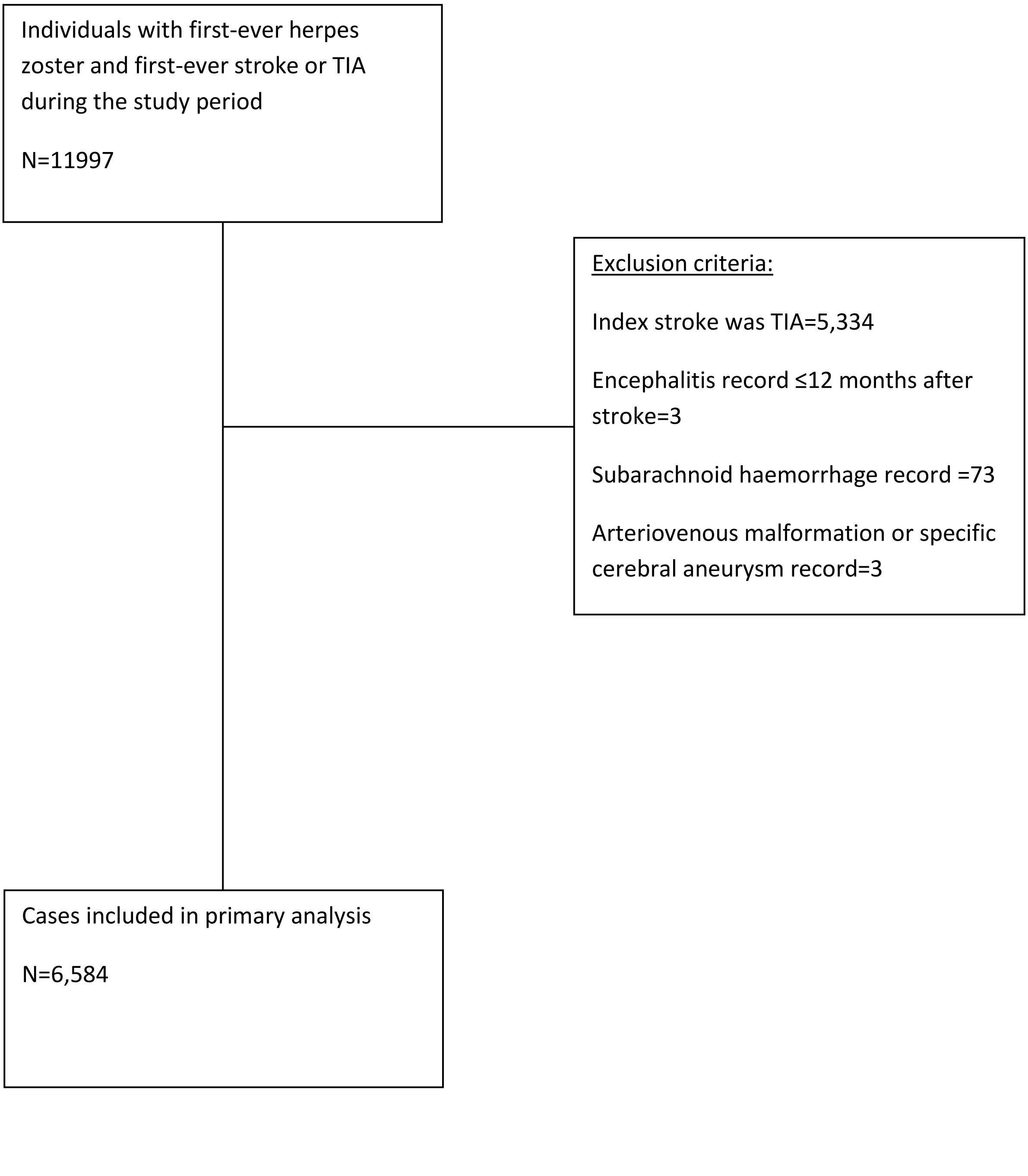

Supplement: Supplementary Data [file supp_ciu098_ciu098supp.pdf]
